# CD1-Restricted T Cells During Persistent Virus Infections: “Sympathy for the Devil”

**DOI:** 10.3389/fimmu.2018.00545

**Published:** 2018-03-19

**Authors:** Günther Schönrich, Martin J. Raftery

**Affiliations:** ^1^Berlin Institute of Health, Institute of Virology, Charité—Universitätsmedizin Berlin, Humboldt-Universität zu Berlin, Berlin, Germany

**Keywords:** human CD1 molecules, antigen presentation, persisting viruses, viral immune evasion, NKT cells

## Abstract

Some of the clinically most important viruses persist in the human host after acute infection. In this situation, the host immune system and the viral pathogen attempt to establish an equilibrium. At best, overt disease is avoided. This attempt may fail, however, resulting in eventual loss of viral control or inadequate immune regulation. Consequently, direct virus-induced tissue damage or immunopathology may occur. The cluster of differentiation 1 (CD1) family of non-classical major histocompatibility complex class I molecules are known to present hydrophobic, primarily lipid antigens. There is ample evidence that both CD1-dependent and CD1-independent mechanisms activate CD1-restricted T cells during persistent virus infections. Sophisticated viral mechanisms subvert these immune responses and help the pathogens to avoid clearance from the host organism. CD1-restricted T cells are not only crucial for the antiviral host defense but may also contribute to tissue damage. This review highlights the two edged role of CD1-restricted T cells in persistent virus infections and summarizes the viral immune evasion mechanisms that target these fascinating immune cells.

## Introduction

The majority of virus infections are self-limiting. Immunocompetent hosts often eliminate the invading pathogen without causing permanent tissue damage. Some viruses, however, can resist clearance, and persist in the host organism in the face of intact antiviral immune responses. Persisting viruses belong to different RNA and DNA virus families and represent a global threat to human health (1). As a strategy for persistence, they utilize chronic infection, latency, or both. Chronic infection is characterized by the continuous generation of infectious virus particles as observed during infection with hepatitis B virus (HBV) (2) or hepatitis C virus (HCV) (3). During latent infection, production of viral progeny is put on ice while the viral genome replicates with the host DNA. In the latent stage, viruses are nearly invisible for the host defense but can reinitiate production of infectious viral particles. Primarily human papillomavirus (HPV) (4) and the human herpesviruses (HHVs) (5) establish latency. Human immunodeficiency virus (HIV) establishes both chronic and latent infection (6). The immunological challenges associated with persisting virus infections are distinct from acute self-limiting infections (7).

Virus persistence is largely the result of an evolutionary arms race between the host immune system and viral immune evasion mechanisms (8). For example, persisting viruses have learned to subvert the attack of cytotoxic T cells, which recognize viral peptides presented by major histocompatibility complex (MHC) class I molecules (9). Cluster of differentiation 1 (CD1) molecules are structurally related to MHC class I heavy chain molecules and also associate with β2-microglobulin (10–12). They are expressed in mammals, birds and reptiles, although isoforms, antigen-binding sites, recycling motifs and genomic locations are not well conserved (13, 14). In striking contrast to MHC class I molecules, CD1 molecules are non-polymorphic and present hydrophobic, primarily lipid antigens to specialized T cells (15, 16). These CD1-restricted T cells are at the frontline of the human immune response against pathogenic microbes including viruses (17). In this review, we discuss lipid-driven T cell responses during persistent virus infections and the corresponding viral counter measures.

## CD1-Restricted T Cells

In humans CD1 molecules are divided into group 1 (CD1a, CD1b, CD1c) and group 2 (CD1d) by sequence homology (18). Group 1 genes can be induced in a coordinated fashion primarily in professional antigen-presenting cells such as dendritic cells (DCs). In contrast, CD1d is constitutively expressed in a wider range of hematopoietic and non- hematopoietic cells (19). CD1-restricted T cells belong to the “unconventional” T cells that do not recognize MHC-bound peptides, and which often show rapid effector functions and orchestrate other immune cells (20–23).

Group 1 CD1-restricted T cells express diverse αβ T-cell receptors (TCRs). They can undergo clonal expansion in the periphery after recognition of stimulatory self-lipids or exogenous lipid antigens derived from bacteria such as *Mycobacterium tuberculosis* (24–26). CD1d-restricted T cells are referred to as natural killer T (NKT) cells because they usually—but not always—express NK1.1 (CD161), a NK cell activating C-type lectin. These cells are further divided into type 1 and type 2 NKT cells.

Type 1 NKT or invariant NKT (iNKT) cells express a semi-invariant αβ TCR, defined by expression of the Vα14-Jα18 TCR in mice and Vα24-Jα18 TCR in humans. They have been analyzed in great detail [recently reviewed in Ref. (17, 27–30)]. The iNKT cells are optimally stimulated by α-galactosylceramide (αGalCer), a glycolipid antigen derived from marine sponge (31). Physiological ligands include cellular and microbial lipids (32). It has been shown that iNKT cells contribute to antiviral responses although the relevant lipid antigens have not yet been defined [recently reviewed in Ref. (33–37)].

Type 2 NKT cells are not stimulated by αGalCer and show much more TCR sequence variability than iNKT cells (38–40). They are more prevalent in humans than iNKT cells, show features of both conventional T cells and iNKT cells and also influence the outcome of infections with persisting viruses (38–41).

### Group 1 CD1-Restricted T Cells

Group 1 CD1-restricted T cells have been analyzed much less intensively than NKT cells. They can be CD4^+^, CD8^+^, or double negative (DN) (42, 43). *In vitro*-studies revealed that after recognition of self or foreign lipid antigens group 1 CD1-restricted T cells become cytoloytic and secrete large amounts of cytokines such as IFN-γ and TNF-α (44–46). These cytokines have a profound antiviral effect and play a pivotal role in controlling persistent virus infections (47). Self-reactive group 1 CD1-restricted T cells are common and the frequency is comparable to alloreactive T cells (estimated to be 1/10 to 1/300 of circulating T cells) (42, 48). They can induce TNF-α dependent DC maturation (49). Intriguingly, self-reactive CD1b-restricted T cells can acquire the phenotype of T helper 17 (Th17) cells and recruit neutrophils (50). The autoreactivity of group 1 CD1-resticted T cells is enhanced by stimulation through pattern recognition receptors (PRRs) (51). It is probable that viruses also trigger activation of self-reactive group 1 CD1-restricted T cells through virus-sensing PRRs and that this contributes to antiviral immunity or virus-induced immunopathogenesis. This notion is supported by the finding that HHV-5, also called human cytomegalovirus (HCMV) can interfere with localization of group 1 CD1 molecules to the cell surface especially CD1b (52).

### CD1d-Restricted T Cells

#### iNKT Cells

As part of the innate immune system iNKT cells perform multiple effector functions rapidly after being activated. Human iNKT cells comprise phenotypically and functionally distinct subsets: CD4^+^, a small fraction of CD4^−^CD8αα^+^ and DN cells (53). The CD4^+^ subset can produce Th2-type cytokines whereas both the CD4^+^ and CD4^−^ subsets can secrete Th1-type cytokines and cytotoxic molecules such as perforin or granzyme B (54–56). The iNKT cells can contribute to both protection from pathogenesis and enhancement of disease (57). In mice, functionally polarized subsets of iNKT cells are generated in the thymus. They secrete Th1-, Th2-, or Th17-like cytokines (NKT1, NKT2, and NKT17 subsets) similar to MHC class II-restricted CD4^+^ T cells and innate lymphoid cells (58–60). In fact, these subsets and the corresponding MHC-restricted T cell subsets share the same transcription factors that regulate their function (61). Further subsets of iNKT cells that are potentially relevant during viral infections have been defined: follicular helper-like iNKT cells (62), IL-9-producing iNKT cells (63), regulatory (Foxp3^+^) iNKT cells (64, 65), and IL-10-producing iNKT cells called NKT10 cells (66).

The iNKT cells can lyse CD1d^+^ target cells, mostly through the interaction between CD95 (Fas) and CD178 (FasL) (67, 68). Virus-infected cells are eliminated by iNKT cells in a CD1d-dependent manner (69). In line with this notion, iNKT cells limit the number of Epstein–Barr virus (EBV)-transformed human B cells *in vitro* by a mechanism requiring direct contact with EBV-infected cells (70). EBV, a HHV that infects more than 90% of the human population worldwide, is associated with tumors, such as Burkitt lymphoma, Hodgkin disease, and lymphomas, in immunosuppressed patients (71). The iNKT cell frequency in human tissue is very low (approximately 0.1% in peripheral blood and spleen) (72). Additionally, in contrast to mice iNKT cells are not enriched in the human liver (73, 74). This suggests that human iNKT cells may be more important in helping orchestrate the antiviral immune response rather than in killing virus-infected cells.

There is ample evidence that iNKT cells attract, stimulate, and regulate other innate cells with antiviral effector functions such as NK cells and neutrophils (21, 22). NK cells are found in most compartments of the human organism at a higher frequency than iNKT cells and are critically involved in protection from persisting viruses (75, 76). The iNKT cells transactivate NK cells through the release of IFN-γ in mice (77–80) or IL-2 in humans (81). Moreover, iNKT cells can directly or indirectly recruit and activate neutrophils. These innate cells contribute to both antiviral defense and virus-induced immunopathogenesis (82–85). NK1.1-negative iNKT lymphocytes can directly recruit neutrophils through preferential IL-17 secretion (86, 87). The iNKT cells can also indirectly promote neutrophil responses by interacting with monocyte-derived DCs resulting in prolonged Ca^+^ influx and release of inflammatory mediators such as PGE_2_ (88). In mice infected with murine cytomegalovirus (MCMV), a well-established model of persistent herpesvirus infection, IL-22 secreted by iNKT cells contributes to recruitment of antiviral neutrophils expressing TNF-related apoptosis-inducing ligand (TRAIL) (85). Moreover, iNKT cells can reverse the suppressive phenotype of neutrophils that is induced by acute-phase reactant serum amyloid A-1 (89). *Vice versa*, neutrophils may reduce inflammatory iNKT cell responses by a contact-dependent mechanism thereby contributing to reduction of inflammation (90). These examples illustrate how intensely iNKT cells and other innate immune cells cross-regulate each other.

Another important antiviral function of iNKT cells is the regulation of adaptive immunity (91). For example, iNKT cells help B cells to proliferate and produce high titers of antibodies (92, 93). B cells present bacterial glycolipids through CD1d to iNKT cells which in turn differentiate into follicular helper-like iNKT cells that provide cognate help to B cells (62, 94–96). Recent evidence suggests that iNKT cells are also critical for production of specific antibodies during viral infections despite the absence of exogenous lipid antigens (97, 98). The underlying mechanism, however, is neither based on follicular helper-like iNKT cell differentiation nor CD1d-mediated cognate B cell help by iNKT cells but on tightly regulated early cytokine secretion by iNKT cells at the interfollicular area (97). On the other hand, iNKT cells licensed by a specialized neutrophil subset restrict activation of autoreactive B cells during inflammasome-driven inflammation through FasL (67).

In addition to helping B cells, iNKT cells promote T cell responses directed against intracellular pathogens such as viruses (79, 99–103). This is accomplished by iNKT-mediated DC maturation, which is of central importance for an efficient antiviral host defense (99, 104–106). It has also been reported that activated CD4^−^CD8αα^+^ iNKT cells curb expansion of antigen-stimulated conventional T cells by CD1d-dependent killing of DCs (107). Thus, iNKT cell subsets regulate adaptive immune responses at different stages.

The importance of crosstalk between iNKT cells and monocyte-derived DCs is underlined by the observation that both cell types are programmed to migrate to inflamed peripheral tissue (55, 108–110). Many persistent viruses such as herpes simplex virus type 1 (HSV-1), HSV-2 and HCMV target monocyte-derived DCs to induce apoptosis or impair antigen presentation through MHC class I molecules (111–113). As a consequence antiviral CD8^+^ T cells have to be activated by uninfected bystander DCs that take up apoptotic debris containing viral antigens and cross-present it through MHC class I molecules (114, 115). This cross-presentation, also called cross-priming, requires pathogen-associated molecular patterns (PAMPs) and/or specialized subsets of Th cells that mature or “license” the cross-presenting DCs (116). It has been shown that uninfected DCs can be licensed for cross-priming by iNKT cells (117). On the other hand, iNKT cells suppress CD8^+^ T cell responses to viral proteins expressed in skin epithelial cells by blocking cross-priming in the draining lymph nodes (118). This suppressive iNKT cell effect may increase the persistence of viruses such as HPV and HSV-1 in the skin. The effect of iNKT cells on CD8^+^ T cell priming (activating versus inhibitory) may be dependent on the type of antigen and the requirement for CD4^+^ T cell helper epitopes (118).

A recent study has shown that iNKT cells release the brake on the adaptive immune response against influenza virus by interfering with myeloid-derived suppressor cells (MDSCs) (119). MDSCs are in fact a heterogeneous population of myeloid progenitor cells including immature DCs, immature macrophages, and granulocytes (120). The iNKT cells abolish the suppressive activity of MDSCs in a crosstalk that requires CD1d and CD40. The finding by De Santo et al. is important as MDSCs facilitate the development of persistent virus infections by impairing antiviral functions of T cells, NK cells, and APCs (121).

#### Type 2 NKT Cells

Evidence that type 2 NKT cells play a protective role in virus infections came from a study that compared virus-induced disease severity between wild-type mice, Jα18 KO mice (lacking only type 1 NKT cells) and CD1d KO mice (lacking both type 1 and type 2 NKT cells) (122). In comparison to iNKT cells little is known about the function of type 2 NKT cells due to the lack of suitable animal models and the difficulty in tracking this poorly defined cell type *in vivo* (41). However, there is evidence that type 2 NKT cell subsets with distinct functional profiles exist (41, 123).

## Clinical Observations

A number of clinical observations suggest involvement of CD1-restricted T cells in either the control of persisting viruses or virus-induced immunopathogenesis.

### Group 1 CD1-Restricted T Cells

**Figure 1:**
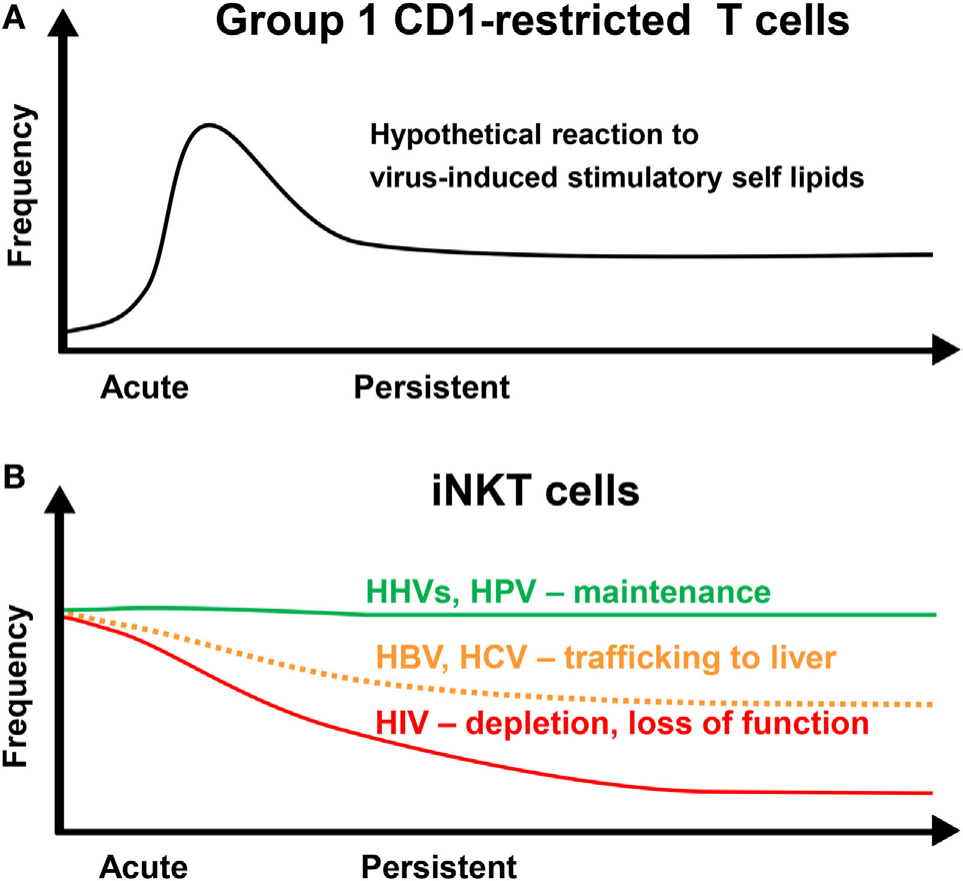
Dynamics of CD1-restricted T cells after persistent virus infections. **(A)** Group 1 CD1-restricted T cells are thought to undergo clonal expansion in response to an acute infection followed by a contraction phase leaving an increased population of memory cells. **(B)** The frequency of invariant NKT (iNKT) cells in the blood is maintained at a stable level over time in individuals infected with human herpesviruses (HHVs) or human papillomavirus (HPV). Human immunodeficiency virus (HIV) infection results in a loss of iNKT cells and a decrease in functionality. Hepatitis B virus (HBV) and hepatitis C virus (HCV) may result in a decrease in iNKT cell frequency due to redistribution into the liver.

Group 1 CD1-restricted T cells from patients with active tuberculosis expand after reexposure to cognate antigen similar to adaptive MHC-restricted T cells (124). In HIV-infected patients, CD1b-restricted T cells recognizing mycobacterial glycolipids are strongly reduced (125). The reduced frequency of mycobacteria-specific CD1b-restricted T cells may contribute to the increased incidence of tuberculosis in this group. The kinetics of group 1 CD1-restricted T cells stimulated by virus-induced self-lipids is not understood. Self-reactive group 1 CD1-restricted T cells show an adaptive-like population dynamics (42). It is possible but has not yet been proven that group 1 CD1-restricted T cell reacting to virus-induced stimulatory self-lipids expand in a similar way (Figure 1A). On the other hand, self-reactive CD1b-restricted T cells have been described that are more like innate T cells (126). These cells may contribute to early antiviral host defense similar to iNKT cells.

### CD1d-Restricted T Cells

Patients with defective iNKT cell responses suffer from severe infection with HHVs (127). For example, boys with X-linked lymphoproliferative disorder have severely impaired iNKT cell development and T cell function that results in life-threatening lymphoproliferation after primary infection with EBV or Kaposi’s sarcoma-associated herpesvirus (KSHV) (128–131). A similar disease was observed in girls with a homozygous mutation in the IL-2-inducible T cell kinase (*ITK*) gene on chromosome 5q31–5q32 (132). Moreover, patients with profound iNKT cell deficiency develop severe VZV-associated disease after vaccination with the live attenuated varicella vaccine (133, 134). Hermansky–Pudlak syndrome type 2, an autosomal recessive disease caused by mutations of the AP3B1 gene, encoding for the beta3A subunit of AP-3, also show reduced numbers of iNKT cells (135). Intriguingly, iNKT cell numbers decrease regardless of the HIV-status in patients with multicentric Castleman disease (MCD), a rare polyclonal lymphoproliferative disorder that is associated with KSHV (136). This observation suggests that a deficiency in iNKT cells contributes to the pathogenesis of KHSV-associated MCD. Although deficient iNKT cells can be regarded as the common denominator of these primary immundeficiencies it has to be kept in mind that other immune cells are affected as well.

In humans with an intact immune system, distinct dynamics of circulating iNKT cells are observed after infection with different persisting viruses. The frequency of peripheral blood iNKT cells after infection with persisting viruses that establish latent infection such as HHVs and HPV remain unaltered (Figure 1B) (98). In contrast, it has been reported that the frequencies of circulating iNKT cells are significantly decreased during chronic HBV and HCV infection and recover during antiviral therapy (Figure 1B) (137, 138). This decrease may be due to trafficking of iNKT cells to the liver as intrahepatic enrichment of iNKT cells is observed in patients with chronic viral hepatitis (137). In another study, however, iNKT cell frequencies between patients with chronic viral hepatitis and healthy individuals were not different (139). CD1d is strongly upregulated on hepatocytes and other liver cells during chronic viral hepatitis (137, 140). In fact, CD1d-restricted T cells contribute to virus-induced liver injury by killing hepatocytes and production of pro-inflammatory cytokines, which promote liver fibrosis and cirrhosis (74, 137, 141–144).

Several studies have shown that iNKT cells are depleted during HIV infection (145–152) (Figure 1B). The remaining iNKT cells display an exhausted phenotype with reduced functionality that may improve with early antiretroviral therapy (152–157). In fact, the level of iNKT cell activation in HIV-infected patients is associated with disease progression markers and the number and functionality of iNKT cells are preserved in non-progressive HIV infection (155, 158). Intriguingly, both CD4^+^ and CD4^−^ iNKT cells are depleted in HIV-infected individuals (149) although CD4^−^ iNKT cells are resistant to HIV infection (148, 150). This could be due to apoptosis of uninfected bystander cells. Triggering of both extrinsic and intrinsic death pathways in T cells due to systemic immune activation has been described in many studies (159).

## CD1-Restricted T Cells in Animal Models of Persistent Virus Infections

### Group 1 CD1-Restricted T Cells

The function of group 1 CD1-restricted T cells has proven difficult to address *in vivo* as laboratory mice do not express group 1 CD1 molecules (160–162). In contrast, mice with a humanized immune system express human group 1 CD1 molecules (163) and open up a new avenue for studying human immune responses to viral pathogens (164). Similarly, human group 1 CD1 transgenic mice have recently been developed (126, 165, 166). Future studies have to investigate a possible role of group 1 CD1-restricted T cells in persisting virus infections by using suitable animal models and tetramers.

### CD1d-Restricted T Cells

The protective and pathogenic role of CD1d-restricted T cells has been investigated in several animal models of persistent virus infections (see Table 1).

**Table 1:** Studies of CD1-restricted T cells in animal models.

Virus	Animal model	Key findings	Reference
HSV-1	CD1d KO and Jα18 KO mice	Type 1 and type 2 NKT cells contribute to virus control and reduce lethality, neuroinvasion, size of lesions and levels of latency	(167, 168)
HSV-1	Jα18 KO mice	iNKT cells are required for an IFN-γ driven subtype profile of HSV-1 specific antibodies	(98)
HSV-2	CD1d KO mice	CD1d KO mice develop a higher disease core after intravaginal HSV-2 infection	(169)
HSV-2	C57BL/6 mice	Intravaginal pre-treatment of C57BL/6 mice with αGalCer lowers the disease core and vaginal viral load	(170)

MCMV	Jα18 KO mice	C57BL/6 mice lacking iNKT cells can control MCMV replication	(172, 220)
MCMV	Jα18 KO mice	iNKT cells contribute significantly to innate control of MCMV in BALB/c mice	(176)
MCMV	CD1d KO and Jα18 KO mice	NKT cells counteract MCMV-induced meyelosuppression	(173)

HBV	HBV transgenic mice	αGalCer-induced release of IFN-γ and type I IFN from iNKT cells stops HBV replication in the liver	(80)
HBV	HBV transgenic mice	Type 2 NKT cells are activated by HBV replication causing hepatitis	(181)
HBV	HBV transgenic mice	Blockade of NKG2D prevents hepatitis mediated by type II NKT cells	(182)
HBV	Ad-HBV infected mice	Type 2 NKT cells control HBV infection through sensing of HBV-induced self-lipids	(180)

SIV	Asian macaques	SIV-induced NKT cell depletion inversely correlates with viral load	(184)
SIV	Asian macaques and sooty mangabeys	Dysfunctionality of NKT cells is associated with increased immune activation and loss of CD4^+^ T cells	(185)

*Ad-HBV, HBV-expressing adenoviral particles; HBV, hepatitis B virus; HSV-1, herpes simplex virus type 1; HSV-2, herpes simplex virus type 2; MCMV, mouse cytomegalovirus; SIV, simian immunodeficiency viruses; NKT, natural killer T; iNKT, invariant NKT*.

#### Herpesviral Infection

Studies in CD1d KO and iNKT cell-deficient (Jα18 KO) mice indicate that CD1d-restricted T cells have a protective role after infection with HSV-1 (98, 167, 168) or HSV-2 (169). In addition, intravaginal pretreatment of C57BL/6 mice with αGalCer lowers HSV-2 disease and inhibits viral replication (170). One report did not confirm an antiviral role for NKT cells, most likely because mice were infected with a less virulent HSV-1 strain (171).

In mice infected with MCMV, activation of iNKT cells by exogenous αGalCer reduces viral replication (172). Furthermore, it has been shown in the MCMV model that iNKT cells can protect from MCMV-induced myelosuppression (173). In C57BL/6 mice NK cells can compensate for iNKT deficiency (172, 174, 175) whereas in BALB/c mice NK cells are less well activated by MCMV and thus iNKT deficiency results in higher viral loads (176).

#### Chronic Viral Hepatitis

In contrast to HHVs, HBV, and HCV infect target cells without causing cytopathic effects. Injection of αGalCer into HBV transgenic mice activates intrahepatic resident iNKT cells and NK cells resulting in noncytopathic control of HBV replication through secretion of type I and II IFN (80, 177). Hepatocytes directly control iNKT cell homeostasis through modulating the balance between activating and non-activating lipids presented by CD1d molecules (178). Accordingly, iNKT cells act as an early warning system for HBV infection, which profoundly alters lipid metabolism (179). For example, type 2 NKT cells recognize CD1d-bound lysophospholipids, antigenic self-lipids that are generated in hepatocytes by a HBV-induced secretory phospholipase (180). The resulting activation of type 2 NKT cells also leads to IL-12 mediated transactivation of iNKT cells and plays a pivotal role in virus control (180). However, type 2 NKT cells can also cause liver injury in HBV transgenic mice (181, 182). It has to be kept in mind that there are major differences between human and murine liver NKT cells when extrapolating these results to humans (73, 74). Nevertheless, human NKT cell lines are stimulated in a CD1d-dependent manner by human hepatocytes (180). Taken together, these findings support clinical observations suggesting a role for CD1d-restricted T cells in antiviral immunity and virus-induced immunopathogenesis in human liver.

#### HIV Infection

In a humanized mouse model of HIV-1 infection activation of type 2 NKT cells inhibits viral replication and prevents virus-induced pancytopenia (183). In Asian macaques, which develop acquired immunodeficiency syndrome (AIDS) during persistent simian immunodeficiency virus (SIV) infection, NKT cell depletion inversely correlates with viral load (184). Comparative studies of Asian macaques and sooty mangabeys, which serve as a natural host and do not develop AIDS during persistent SIV infection, suggest that NKT cells protect from SIV-induced immune activation and immunodeficiency (185).

## Activation of CD1-Restricted T Cells

In contrast to other microbes such as bacteria, viruses have not been investigated with regard to CD1 ligands, although viral infection can stimulate CD1-restricted antiviral T cells. Activation of CD1-restricted T cells during viral infection could be triggered by CD1 molecules presenting antigenic self-lipids. In addition, CD1-independent mechanisms that activate CD1-restricted T cells such as cytokine release have been described.

### Group 1 CD1-Restricted T Cells

Autoreactive group 1 CD1-restricted T cells are stimulated by DCs that express group 1 CD1 molecules and present increased amounts of antigenic self-lipids upon activation by bacterial PAMPs through TLRs (186, 187). It is very likely but has not yet been shown that group 1 CD1-restricted autoreactive T cells are activated in a similar fashion during viral infections upon activation of DCs through virus-sensing PRRs.

### CD1d-Restricted T Cells

#### CD1d-Dependent Activation

The limited repertoire of microbial and endogenous ligands presented by CD1d is expanding (188–190). However, the identity of self-lipids presented during viral infection is still enigmatic. Stimulatory self-lipids could be channeled into the CD1d-restricted antigen presentation pathway by several distinct mechanisms. First, there is ample evidence that TLR stimulation increases CD1d presentation of antigenic self-lipids resulting in iNKT cell activation (191–197). Besides modulating the repertoire of self-lipids, viruses also increase CD1 surface expression through triggering PRRs (198). Second, viral invasion and inflammation is associated with pathological hypoxia that activates hypoxia-inducible factor (HIF), a “master regulator” of adaptive immune responses (199, 200). HIF alters lipid metabolism in such a way that self-reactive NKT cells are activated (201, 202). Third, viruses profoundly rewire host lipid metabolism and remodel lipid distribution to boost in a coordinated fashion viral entry, replication, assembly, and egress (203–208). Disturbance of the normal lipid trafficking patterns within the cell due to enveloped virus production (HHVs, HBV, HCV) or even naked virus (HPV) will allow access of otherwise sequestered lipids or altered lipids to the relevant CD1 presenting compartment. Furthermore, after assembly and release from the infected host cell the envelope of the newly built viral particle may carry the ligand with it and deliver it during infection to another host cell. Taken together, several distinct mechanisms facilitate CD1d-dependent iNKT cell activation during different stages of the viral life cycle.

Of note, viral immune evasion mechanisms targeting other immune components may alert iNKT cells. For example, MHC class I downregulation by virus-encoded immunevasins induces CD1d upregulation and enhances NKT cell activation (209). This finding is supported by other observations. First, low pH stripping of MHC class I molecules augments CD1d surface expression and activates iNKT cells (210). Second, APCs from transporter associated with antigen presentation 1 (TAP1)-deficient mice are defective in MHC class I antigen presentation but show an increased capacity to stimulate iNKT cells (210, 211). This reverse correlation between CD1d and MHC class I surface expression may enable iNKT cells to detect the loss of MHC class I molecules, a situation called “missing self” (212). The underlying mechanism is not yet fully understood but may involve masking of CD1d by MHC class I molecules on the cell surface (210). Increased iNKT cell activation during persistent virus infections may also result from viral blockers inhibiting the autophagic machinery, which downregulates iNKT cell responses through CD1d internalization (213, 214).

#### CD1d-Independent Activation

CD1d-restricted T cells can also be stimulated independently of the TCR. First, there is ample evidence that pro-inflammatory cytokines (IL-12, IL-18, or type I IFN) released from APCs after stimulation through PRRs activate iNKT cells predominantly in a CD1d-independent manner (215, 216). It may be that an initial CD1d-mediated TCR signal is still required (217). CD1d-independent iNKT cell activation was observed in mice infected with MCMV, and was driven by type I IFN and IL-12 after DC stimulation (176, 218–220). The factors that determine whether pro-inflammatory cytokines are sufficient for activation of antiviral iNKT cells are unclear.

Second, engagement of NKG2D on NKT cells results in CD1d-independent activation and subsequent killing of ligand-expressing target cells (182, 221). NKG2D is an activating receptor expressed on CD4^−^ iNKT cells and is triggered by stress ligands. The latter are upregulated during viral infections after stimulation of virus-sensing PRRs such as RIG-I and MDA-5 (222, 223). In addition, NKG2D can also co-stimulate TCR-mediated activation of CD4^−^ iNKT cells (221).

Third, iNKT cells can be activated by apoptotic cells through TIM-1, a member of the T-cell immunoglobulin mucin (TIM) family of cell surface proteins (224). TIM-1 is constitutively expressed by NKT cells and serves as a receptor for phosphatidylserine, an important marker of cells undergoing apoptosis (225). This mode of iNKT cell activation may be relevant during persistent virus infections because viruses frequently drive their host cells into apoptosis (226).

## Viral Evasion of CD1-Restricted T Cells

During the evolutionary arms race with the host immune system persisting viruses have developed multilayered defense strategies to interfere with antiviral CD1-restricted T cells (17, 33, 35, 36, 227). They target the CD1 antigen presentation machinery either on the transcriptional, posttranscriptional, or posttranslational level.

### Group 1 CD1-Restricted T Cells

The group 1 CD1 molecules are expressed on professional APCs in response to certain cytokines such as granulocyte-macrophage colony-stimulating factor (228). The anti-inflammatory cytokine IL-10 prevents upregulation of group 1 CD1 molecules on professional APCs such as monocyte-derived DCs (229–231). In fact, the activity of IL-10 is crucial for establishing viral persistence (232). Large DNA viruses such as herpesviruses have acquired numerous genes from their host including viral homologs of IL-10 (vIL-10) to subvert the host immune response (233, 234). For example, vIL-10 from HCMV and EBV not only prevent upregulation of MHC class I/II molecules but also group 1 CD1 molecules (52, 235). Recent reports suggest that vIL-10 strongly induces the expression of its cellular counterpart in monocyte-derived cells thereby potentiating its immunomodulatory effect (236, 237). In addition, viral proteins with no homology to cellular proteins such as HCMV-encoded pUL11 or EBV-encoded latent membrane protein 1 highjack cellular signaling pathways to induce expression of cellular IL-10 (238, 239).

Group 1 CD1 molecules are downregulated from the cell surface during the early phase of HCMV infection with CD1b being especially sensitive to this effect (52). This block is not performed by known HCMV-encoded MHC class I-blocking molecules and results in the intracellular accumulation of group 1 CD1 molecules (52). The underlying mechanism has not yet been described. In addition, HIV-1 negative factor (Nef) downregulates CD1a after infection of immature DCs with HIV-1 and impairs stimulation of CD1a-restricted T cells (240, 241). In order to modulate host membrane trafficking pathways HIV-1 Nef has to interact with various host proteins through distinct motifs (242). Nef-mediated CD1a downregulation might require the interaction of Nef with hematopoietic cell kinase and p21-activated kinase 2, which are both expressed in immature DCs (241). Collectively, these findings support the idea that group 1 CD1 molecules contribute to lipid-driven antiviral immune responses.

### CD1d-Restricted T Cells

#### Viral Downregulation of CD1d Gene Transcription

**Figure 2:**
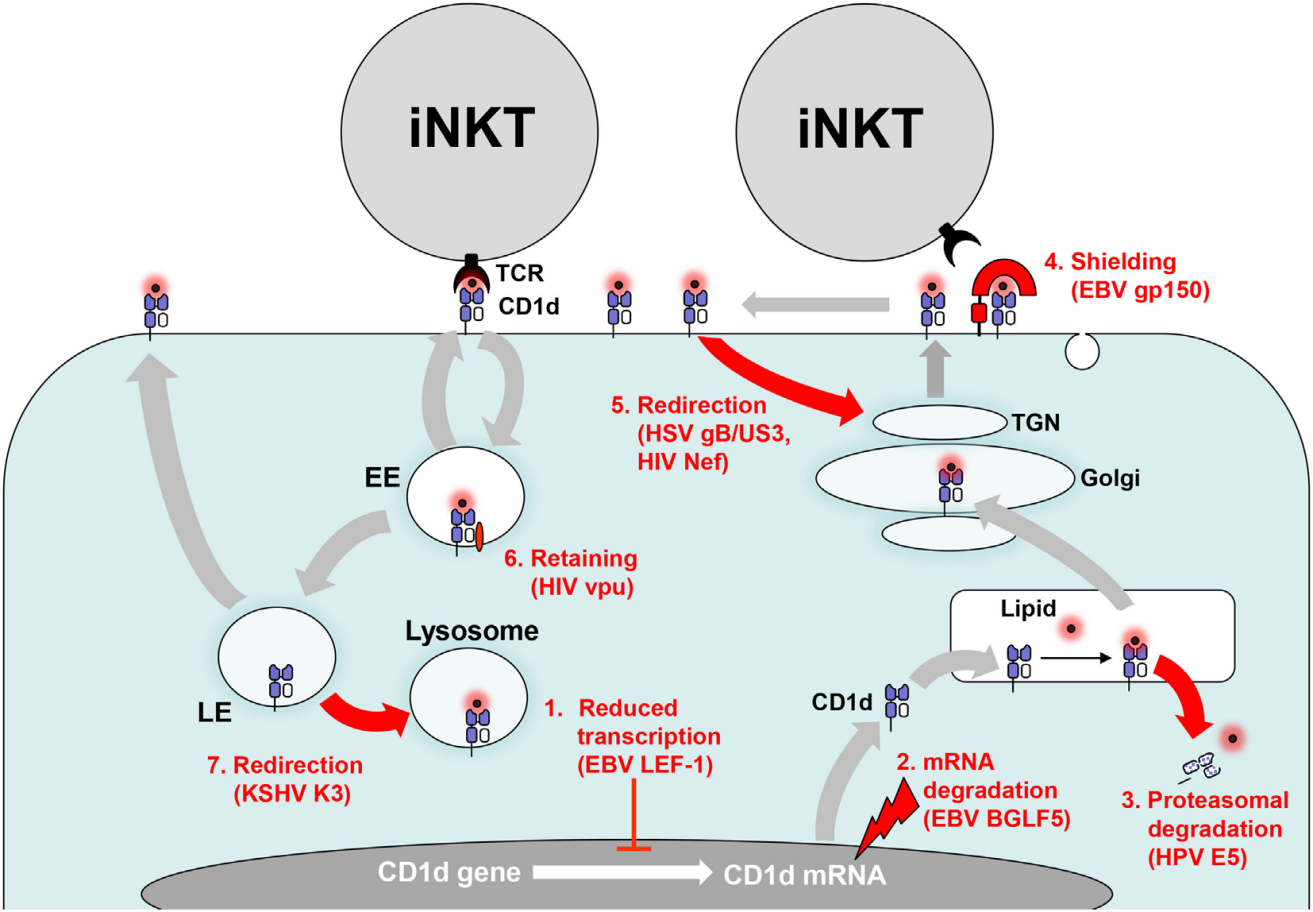
Evasion of CD1d antigen presentation. Persisting viruses evade invariant NKT (iNKT) cell activation by interfering with CD1d biosynthesis and CD1d trafficking. (1) CD1d gene transcription is downregulated by increased binding of Epstein–Barr virus (EBV)-encoded lymphoid enhancer-binding factor 1 (LEF-1) to the distal region of the CD1d promotor. (2) Viral host shutoff factors such as EBV BGLF5, an early lytic phase protein, inhibit protein synthesis by degrading mRNA. (3) Human papillomavirus (HPV) E5 translocates CD1d into the cytosol for proteasomal degradation. (4) EBV gp150 provides an abundantly sialylated glycan shield for CD1d on the cell surface preventing recognition by iNKT cells. (5) Herpes simplex virus type 1 (HSV-1) glycoprotein B/US3 and human immunodeficiency virus (HIV) negative factor (Nef) redirect CD1d to the trans-Golgi network (TGN). (6) In contrast, HIV Nef retains CD1d in the early endosome (EE). (7) Kaposi’s sarcoma-associated herpesvirus (KSHV) K3 redirects CD1d from the late endosome (LE) to the lysosome.

In contrast to group 1 CD1 molecules, CD1d is constitutively expressed not only in myeloid cells such as B cells, macrophages, and DCs and but also in epithelial tissue. Latent EBV infection that is associated with transformation of B cells results in complete shutdown of CD1d mRNA expression and the lack of iNKT cell activation (70). This is due to increased binding of lymphoid enhancer-binding factor 1 to the distal region of the CD1d promotor (Figure 2). Moreover, CD1d transcription is downregulated during severe primary HCMV infection (243).

#### Virus-Induced Degradation of CD1d mRNA

Replicating α- and γ-herpesviruses induce global mRNA degradation to shut off host protein synthesis and reallocate cellular resources to their own need by different mechanisms (244). It has been shown that EBV-encoded shutoff protein BGLF5, a lytic phase protein, downregulates multiple immune components including CD1d (Figure 2) (245, 246).

#### Viral Interference With Trafficking of CD1d Molecules

After translation, CD1 heavy chains bind to β2-microglobulin and are loaded with self-lipids in the endoplasmic reticulum (ER). This trimeric complex travels then to the cell surface through the secretory pathway. Subsequently, a tyrosine motif within their cytoplasmic tail enables human CD1 molecules to bind the μ-subunit of adaptor protein complex (AP)-2 (CD1b-d) and AP-3 (CD1b and murine CD1d). In contrast, CD1a lacks AP-2 and AP-3 sorting motifs. Because of these differences CD1 molecules traffic through distinct intracellular compartments for acquiring lipids and present these lipids to CD1-restricted T cells (247–249). Persistent viruses utilize various posttranslational mechanisms to interfere with antigen presentation through CD1d molecules in different cellular compartments.

#### Virus-Induced Proteasomal Degradation of CD1d in the Cytosol

HPV subverts immune responses through expression of E5 (250). Clinical samples of HPV-infected cervical epithelium show decreased CD1d expression (251). Further analysis of transfected cell lines revealed that HPV E5 interacts with calnexin, a chaperone involved in CD1d folding in the ER, and finally targets CD1d to the cytosolic proteolytic pathway (Figure 2) (251).

#### Viral Shielding of Surface CD1d Molecules

A novel broadly acting mechanism of herpesviral subversion of antigen presentation has been discovered recently (252). EBV-encoded gp150, which is heavily glycosylated and expressed in the late phase of EBV replication, inhibits antigen presentation by MHC class I and II as well as CD1d molecules. The EBV-encoded gp150 does not interfere with recycling of CD1d molecules but prevents their detection by iNKT cells on the cell surface through an abundantly sialylated glycan shield (Figure 2) (252). Of interest, the Ebola virus glycoprotein masks MHC class I molecules by a similar mechanism but an effect on CD1d has not yet been tested (253). Further studies have to elucidate whether other herpesviruses have developed are similar strategy to mask antigen-presenting molecules on the cell surface.

#### Viral Modulation of CD1d Recycling

Persisting viruses can also subvert CD1 antigen presentation by interfering with CD1 recycling. For example, CD1d surface expression is downregulated during productive infection with HHVs such as HSV-1 (209, 254) and KSHV (255). This effect was dependent on the viral titer used for infection of CD1d-expressing cells (209).

Further attempts identified several viral proteins interfering with CD1d recycling. For example, KSHV-encoded modulator of immune recognition proteins 1 and 2 (also known as K3 and K5) function as membrane-bound E3-ubiquitin ligases and reduce expression of a number of immunologically important surface molecules including MHC class I and CD1d (256). In fact, K3 and K5 have been pilfered from the host genome and belong to the *M*embrane *A*ssociated *R*ING-*CH* family of E3 ligase (MARCH) proteins. They reroute both MHC class I and CD1d to the lysosomal compartment by ubiquitinylating a unique lysine residue on the cytoplasmic tail (Figure 2) (255). In contrast to MHC class I, however, the CD1d complexes are resistant to lysosomal degradation resulting in reduced CD1d levels on KSHV-infected B cells although the total cellular amount of CD1d is not altered (255).

A recent study shows that CD1d molecules on the APC surface build nanoclusters and that these structures are important for iNKT cell activation (257). Intriguingly, the actin cytoskeleton prevents CD1d nanoclustering (257). This finding is in accordance with a previous study showing that disruption of actin filaments increases CD1d antigen presentation (258). It may also explain why HSV-1 lacking VP22 (UL49), a viral tegument protein that stabilizes microtubules, lacks the ability to inhibit CD1d recycling (259). Although VP22 is necessary for HSV-1 induced CD1d downregulation, other viral proteins are additionally required. The type II kinesin motor protein KIF3A, which transports proteins along the microtubule network, is necessary for CD1d surface expression (260). Of relevance, KIF3A is phosphorylated by US3, a HSV-1 encoded protein that induces downregulation of CD1d by suppressing its recycling (261). Another HSV-1 protein, glycoprotein B (gB), binds to CD1d within the ER and remains stably associated throughout CD1d trafficking (261). Both US3 and gB seem to be required for relocalization of CD1d to the trans-Golgi network (TGN) thereby reducing CD1d surface expression and iNKT cell activation (Figure 2) (261). In contrast, a previous study has reported that CD1d is trapped in lysome-like structures during HSV-1 infection (254). Similarly, another study reported that CD1d molecules are degraded in the lysosomes after HSV-1 induced phosphorylation of a dual residue motif (T322/S323) in the cytoplasmic tail of CD1d (262, 263). However, under normal conditions CD1d complexes are resistant to lysosomal degradation (255, 264).

HIV-1 encodes two proteins, Nef and viral protein U (Vpu) which target numerous immunologically important surface molecules including CD1d (265). In HIV-1 infected DCs, Vpu neither induces endocytosis nor rapid degradation but suppresses CD1d recycling by retaining CD1d in the early endosome (196, 266, 267). In contrast, Nef increases CD1d internalization and re-localizes CD1d molecules to the TGN (196, 268, 269). Thus, Vpu and Nef interfere with iNKT cell activation by complementary mechanisms (Figure 2). As a result, the capacity of HIV-1 infected DCs to stimulate iNKT cells is impaired (196).

The unique short (US) region of HCMV encodes several proteins including US2 and US11 that interfere with antigen presentation through MHC class I molecules (270). US2 and US11 induce rapid translocation of MHC class I heavy chains from the ER into the cytosol and subsequent degradation by the proteasome (271, 272). US2 and US11 also physically interact with CD1d but do not downregulate CD1d surface expression (273, 274). However, reduced activation of iNKT cells after stimulation with αGalCer-pulsed US2-expressing APCs has been reported but the underlying mechanism is unclear (274).

#### Viral Disruption of the iNKT Cell–DC Axis

The bidirectional interaction between DCs and iNKT cells is crucial for an efficient antiviral immune response (28, 275). During infection triggering of a combination of PRRs stimulates IL-12 release from DCs and increases the presentation of antigenic self-lipids by CD1d (191, 195, 197). In fact, signaling through PRRs inhibits degradation of glyosylceramides in the lysosome thereby increasing the amount of lipids that stimulate iNKT cells (193, 276). Once activated iNKT cells further enhance IL-12 production by DCs through CD40–CD40 ligand stimulation. This amplification loop then results in NK cell transactivation and increased responses of MHC-restricted CD4^+^ as well as CD8^+^ T cell responses. Persisting viruses such as HHVs use several different strategies to blunt DC function (277). For example, VZV interferes with TLR signaling in DCs thereby decreasing IL-12 production (278). Moreover, herpesviral IL-10 homologs can decrease the ability of uninfected DCs to secrete IL-12 (234). Virus-infected DCs that do not produce biologically active IL-12 fail to activate the antiviral functions of iNKT cells (279). Moreover, HCMV-infected DCs upregulate on the surface death ligands such as TRAIL that kill T cells (113). Taken together, it is likely that multilayered viral countermeasures severely impair activation and function of iNKT cells thereby disrupting the important iNKT cell–DC axis.

#### Viral Interference With CD1d-Independent Activation of CD1d-Restricted T Cells

Persistent viruses such as HHVs also interfere with NKG2D ligand upregulation on virus-infected cells thereby reducing the likelihood of NKG2D-mediated activation of antiviral iNKT and type 2 NKT cells (280, 281). Moreover, HSV-1-infected keratinocytes block cytokine-dependent activation of iNKT cells (282).

#### Virus-Induced Functional Impairment of CD1d-Restricted T Cells

Persisting viruses not only target CD1 antigen presentation and interfere with activation of CD1-restricted T cells but can also induce a state of unresponsiveness in CD1-restricted T cells. It has been demonstrated *in vitro* that HSV-1-infected keratinocytes impair TCR signaling in iNKT cells in a contact-dependent manner (282). In contrast to DCs, CD1d is not downregulated on keratinocytes after infection with HSV-1. This finding nicely illustrates that depending on the infected cell type one and the same virus use different strategies to evade CD1d-restricted T cell responses. Moreover, iNKT cells from patients with KSHV-associated MCD, a severe B-cell lymphoproliferative disorder, show impaired proliferation when stimulated with αGalCer (136). This hyporeactive state is indicative of virus-induced iNKT cell anergy or exhaustion. In accordance, induction of anergy in iNKT cells has been described in many reports and is required for prevention of uncontrolled inflammation and tissue destruction (283). On the other hand, it also allows viruses to replicate and spread more efficiently. The underlying mechanisms of virus-induced iNKT cell anergy are unknown but may involve viral modulation of costimulatory molecules and other signaling molecules on APCs (284).

Recently, a regulatory iNKT cell subset called NKT10 has been described that bear similarities to Tregs (66). Tregs suppress protective immune responses thereby supporting virus persistence and at the same time also reducing the inflammation-mediated tissue damage (285). The NKT10 cells occur naturally in mice and humans and expand after stimulation (66). It would be interesting to investigate whether persisting viruses increase the frequency and activity of NKT10 cells to curb antiviral immune responses.

## Conclusion and Future Directions

The risk for the host in terms of severe disease from a persistent virus infection ranges from low in case of well-adapted viruses such as the HHVs to ineluctable in case of a recent émigré such as HIV-1. CD1-reactive cells play a significant role in this process. Although the role of self-reactive NKT cells in antiviral immunity and immunopathogenesis has been analyzed in the clinical setting and in experimental models, it is unclear how group 1 CD1-restricted T cells contribute to antiviral immunity. Future efforts should define in detail the antigenic self-lipids that are induced and loaded on CD1 molecules during viral infections and how they program antiviral immunity. This knowledge can then be harnessed to develop novel vaccines and adjuvants for protection from persistent virus infection.

## Author Contributions

Both authors contributed to the conception, writing, and critical revising of this review.

## Conflict of Interest Statement

The authors declare that the research was conducted in the absence of any commercial or financial relationships that could be construed as a potential conflict of interest.
